# Supplementary material for: Oxidized LDL‐dependent pathway as new pathogenic trigger in arrhythmogenic cardiomyopathy
Source: EMBO Mol Med. 2021 Aug 2;13(9):e14365. doi: 10.15252/emmm.202114365 (PMC8422076; doi:10.15252/emmm.202114365)
Supplement: Supplementary file 1 — Appendix [file EMMM-13-e14365-s004.pdf]

Appendix

Oxidized LDL-dependent pathway as new pathogenic trigger in Arrhythmogenic Cardiomyopathy.

Table of content:

Supplemental Results

Appendix Figure S1

Dataset EV1

Appendix Figure S2

Appendix Figure S3

Appendix Figure S4

Appendix Figure S5

Appendix Figure S6

Appendix Figure S7

Appendix Table S1

Appendix Table S2

Appendix Table S3

Appendix Table S4A and S4B

Appendix Table S5

Appendix Table S6

Appendix Table S7

Appendix Table S8

Supplemental Results:

Investigation of oxidative stress and dyslipidemia as genetically determined secondary traits.

The comparison of plasma oxLDL levels between ACM patients with overt ACM phenotype carrying ACM-related causative mutations and their relatives, carriers of the same mutations but not clinically affected by the pathology, indicates a higher amount of oxLDL in the former (**Figure1B**). This result suggests that the main causative ACM mutation may not be responsible for augmented oxLDL. Thus, we investigated a genetic predisposition for oxidized lipid increase in ACM affected patients.

We performed the exome sequencing on three families with low ACM penetrance, in which causative *PKP2* mutations were identified (**Appendix Figure S1**, **Dataset EV1**). We analysed the presence and the co-segregation with the ACM phenotype of variants in a selected panel of genes associated with dyslipidemia and oxidative stress (**Dataset EV1**), with two different analysis criteria, either cumulative of the three families (**Appendix Table S4A**), or separately in each family (**Appendix Table S4B**). The retrieval of 14 such variants introduces the concept of a possible influence of the genetic background on ACM lipid and oxidative status.

ACM patient genetics do not influence oxLDL plasma levels. Analysis on the whole cohort.

The whole cohort of ACM patients was subjected to genetic analysis of ACM-associated genes. Results are reported in **Appendix Table S5**. To confirm the data of **Figure1A** on the whole cohort of ACM patients, we performed the comparison of oxLDL plasma levels between HC and our whole cohort, finding a significant difference in oxLDL concentrations (n=36 vs. n=65; HC 66.74±5.79 vs. ACM 213.5±41.81ng/ml; p=0.009; **Appendix Figure S2A**). In addition, comparing oxLDL plasma levels between *PKP2*-mutated ACM patients vs. ACM patients with other desmosomal, non-desmosomal mutations and gene elusive of our cohort of 65 genotyped patients, we did not detect differences (**Appendix Figure S2B, S2C**), indicating that oxLDL plasma concentrations are not linked to the patients' genotype of our cohort.

Comparison of the mass of different lipid classes accumulated during adipogenic differentiation.

We performed a quantitative and qualitative characterization of C-MSC lipid profile after 7 days of adipogenic differentiation. Lipidomic assays revealed higher mass in ACM vs. HC C-MSC of all analysed lipid classes

(**Appendix Figure S3**). In particular, ACM cells accumulated significantly more free cholesterol (n=5 vs. n=4; μg free cholesterol/10^6 cells ACM 11.95 ± 1.05 vs. HC 8.24 ± 1.15; p = 0.05) and triglycerides than HC C-MSC (n=5 vs. n=4; μg triglycerides/10^6 cells ACM 35.23 ± 5.38 vs. HC 18.64 ± 3.22; p = 0.04; **Appendix Figure S3A**). These data are in line with the pictures of ACM C-MSC showing neutral lipid accumulation detected through Oil Red O (ORO) staining. Indeed, we found a linear correlation between triglyceride quantity and ORO staining levels (**Appendix Figure S3B**). Triglycerides accumulation confirmed the ongoing transition of C-MSC toward an adipogenic lineage.

Susceptibility to oxLDL treatment of ACM C-MSC upon PKP2 expression.

To unravel the reasons of higher susceptibility to oxLDL treatment of ACM C-MSC, we evaluated its dependency upon PKP2 expression. Indeed, most of our patients carry a genetic mutation in the PKP2 gene, leading to its haploinsufficiency. Moreover, we previously demonstrated a lower expression of PKP2, even in ACM patients without a known causative mutation (Sommariva et al, 2016). To this purpose, we silenced PKP2 in HC cells, using lentiviral particles containing PKP2 shRNA. A reduction of PKP2 to half of its original expression (**Appendix Figure S4**) significantly increased lipid accumulation in response to oxLDL (n = 3 each; ORO quantification PKP2 shRNA AM 4.11 ± 0.09 vs. PKP2 shRNA AM+oxLDL 7.76 ± 0.67; p = 0.05; **Appendix Figure S4**), whereas no changes in lipid accumulation were detected between scrambled control cells cultured in AM or AM+oxLDL. This indicates that susceptibility to oxLDL treatment is dependent upon PKP2 expression levels. As previously demonstrated in (Sommariva et al, 2016), we also confirmed that HC C-MSC can accumulate lipid when PKP2 expression is reduced (n = 3 each; ORO quantification scramble AM 1.00 ± 0.04 vs. PKP2 shRNA AM 4.11 ± 0.09; p = 0.001; **Appendix Figure S4**).

Lipid accumulation and CD36 levels increase in ACM hiPSC-CM upon PPARγ agonism

To understand if oxLDL/CD36/PPARγ circuitry is potentially activated also in human ACM cardiomyocytes, CD36 membrane expression was evaluated in hiPSC-CM derived from ACM patients and unaffected relatives after adipogenic stimulation. While limited lipid accumulation was detected in GM, in analogy to what observed in C-MSC, CD36 expression showed a trend of increase in ACM hiPSC-CM than in controls. When exposed to AM containing the PPARγ agonist rosiglitazone, ACM hiPSC-CM exhibited a higher content of

intracellular lipids compared to HC hiPSC-CM, as previously reported (25) (relative BODIPY staining ACM hiPSC-CM 24.02±4.94 vs. HC hiPSC-CM 10.38±1.94; p=0.01; **Appendix Figure S5A**). AM+rosiglitazone treatment led to CD36 expression increase both in HC (MFI 6.21±1.59, p=0.004 vs. GM) and ACM hiPSC-CM (MFI 6.10±0.94, p=0.01 vs. GM; **Appendix Figure S5B**). As expected, the increased amount of membrane CD36 after AM+rosiglitazone treatment determined higher oxLDL internalization in ACM hiPSC-CM (ACM DiI oxLDL internalization GM 0.53±0.09 vs. AM+rosiglitazone 3.70±0.25; p=0.009; **Appendix Figure S5C**).

Characterization of *Pkp2*+/- model in CD.

To validate our hypothesis on an ACM *in vivo* model, we took advantage of the well-established *Pkp2+/-* mouse (Grossmann et al, 2004). In *Pkp2*+/- hearts we found similar levels of lipid accumulation, despite an increase of PPARγ immunoreactive signal in ACM mice when compared to wild type (WT) mice (n=9; PPARγ densitometric sum/nuclei number WT 1±0.36 vs. *Pkp2*+/- 5.83±0.90; p=0.0001). Comparable expression of MDA and CD36 were found in the two strains (**Appendix Figure S6A-D**). Moreover, at 2D echocardiography, RV and LV functions were similar in the two strains (**Appendix Figure S6D**).

In analogy with human C-MSC, isolated *Pkp2*+/- mouse C-MSC cultured in AM for 6 days, accumulated higher neutral lipids than WT C-MSC (n=5; ORO relative lipid accumulation *Pkp2*+/- 34.61±9.23 vs. WT 1±0.43; p=0.007; **Appendix Figure S7**), indicating that mouse *Pkp2*+/- C-MSC are genetically predisposed to lipid accumulation.

Validation by Western Blot of the quantification of the immunofluorescence analyses performed on mouse hearts.

In order to validate the quantification of PPARγ, MDA, and CD36 expression calculated by immunofluorescence, we performed the Western Blot analysis of proteins from murine total heart tissue lysates. In accordance to the results obtained with immunofluorescence, we found a higher expression of PPARγ in *Pkp2*+/- mice when compared to WT already in CD (n=3 vs. n=4; PPARγ/GAPDH *Pkp2*+/- CD 22.71±9.70 vs. WT CD 1.00±0.30; p=0.03; **Appendix Figure S9B**). Moreover, HFD feeding provoked a substantial increase of PPARγ expression in ACM mice (n=3; PPARγ/GAPDH *Pkp2*+/- HFD 40.75±5.25 vs.

WT HFD 20.26±2.40; p=0.03; **Appendix Figure S9B**). *Pkp2*+/- mouse hearts also showed oxidative stress (n=3; MDA/GAPDH *Pkp2*+/- HFD 39.25±9.58 vs. WT HFD 11.88±2.27; p=0.001; **Appendix Figure S9B**) and CD36 level increase (n=3; CD36/GAPDH *Pkp2*+/- HFD 20.11±1.17 vs. WT HFD 15.60±1.43; p=0.04; **Appendix Figure S9C**) after HFD if compared to WT.

In addition, we confirmed that atorvastatin treatment significantly reduced PPARγ (n=3; PPARγ/GAPDH *Pkp2*+/- HFD 40.75±5.25 vs. *Pkp2*+/- HFD+atorva 4.63±2.35; p=0.0009; **Appendix Figure S9C**), MDA (n=3; MDA/GAPDH *Pkp2*+/- HFD 39.25±9.58 vs. *Pkp2*+/- HFD+atorva 1.27±0.70; p=0.005; **Appendix Figure S9C**), and CD36 expression (n=3; CD36/GAPDH *Pkp2*+/- HFD 20.11±1.17 vs. *Pkp2*+/- HFD+atorva 0.48±0.13; p<0.0001; **Appendix Figure S9C**).

HFD administration leads to electrical dysfunction in *Pkp2+/-* mice

The analysis of surface ECG of the mouse groups demonstrated no differences in ECG parameters between WT and *Pkp2+/-* mice fed a CD, while *Pkp2+/-* mice fed a HFD showed QRSp widening (n=10 vs. n=9; QRSp *Pkp2*+/- HFD 18.80±0.43 vs. *Pkp2*+/- CD 16.22±0.33 msec; p=0.0001; **Appendix Figure S11A**), fragmented and prolonged terminal activation duration (n=10 vs. n=9; TAD *Pkp2*+/- HFD 9.24±0.43 vs. *Pkp2*+/- CD 7.25±0.27 msec; p=0.0031; **Appendix Figure S11A**) and QRS amplitude decrease (n=10 vs. n=9; QRS amplitude *Pkp2*+/- HFD 829.50±59.65 vs. *Pkp2*+/- CD 1414.00±119.80 mV; p=0.0007; **Appendix Figure S11A**), when compared to control groups. All these features have been described in ACM patients ECG (Cox et al, 2009; De Lazzari et al, 2018; Peters et al, 2008; Zhang et al, 2014). Since the *Pkp2+/-* HFD group defects in the ECG traces are indicative of right ventricular conduction delay, and since ventricular conduction velocity is related to connexin-43 (CX43) expression and localization (Fontes et al, 2012), we analysed mouse cardiac tissue by CX43 immunofluorescence. Indeed, ECG changes were substantiated by decreased expression and mislocalization of CX43 in *Pkp2+/-* HFD mice (n=10 vs. n=9; CX43 *Pkp2*+/- HFD 0.47±0.14 vs. *Pkp2*+/- CD 1.12±0.15; p=0.0091; **Appendix Figure S11B**).

Atorvastatin administration prevents ACM electrical changes in HFD-fed *Pkp2+/-* mice

To evaluate the pharmacological effect of atorvastatin on the electrical changes seen in **FigureS11**, the ECG traces of *Pkp2+/-* mice were fed a 3-month HFD supplemented with 20mg/kg atorvastatin were analysed. We found that atorvastatin was able to restore to pre-diet levels of QRS duration (n=10 vs. n=9; QRSp *Pkp2*+/- HFD 18.80±0.43 vs. *Pkp2*+/- HFD+atorva 14.00±0.44 msec; p<0.0001; **Appendix Figure S12A**), TAD (n=10 vs. n=9; TAD *Pkp2*+/- HFD 9.24±0.43 vs. *Pkp2*+/- HFD+atorva 5.80±0.39 msec; p<0.0001; **Appendix Figure S12A**) and QRS amplitude (n=10 vs. n=9; QRS amplitude *Pkp2*+/- HFD 829.50±59.65 vs. *Pkp2*+/- HFD+atorva 1247.00±108.10 mV; p=0.0029; **Appendix Figure S12A**). Fragmentation of the terminal QRS was not evident any more (**FigureS12A**). Accordingly, CX43 levels were increased respect to *Pkp2+/-* mice with the sole HFD (n=10 vs. n=9; CX43 *Pkp2*+/- HFD 0.47±0.14 vs. *Pkp2*+/- HFD+atorva 1.07±0.25; p=0.049; **Appendix Figure S12B**), and CX43 was manly localized at intercalated disks (**FigureS12B**).

Supplemental Figures:

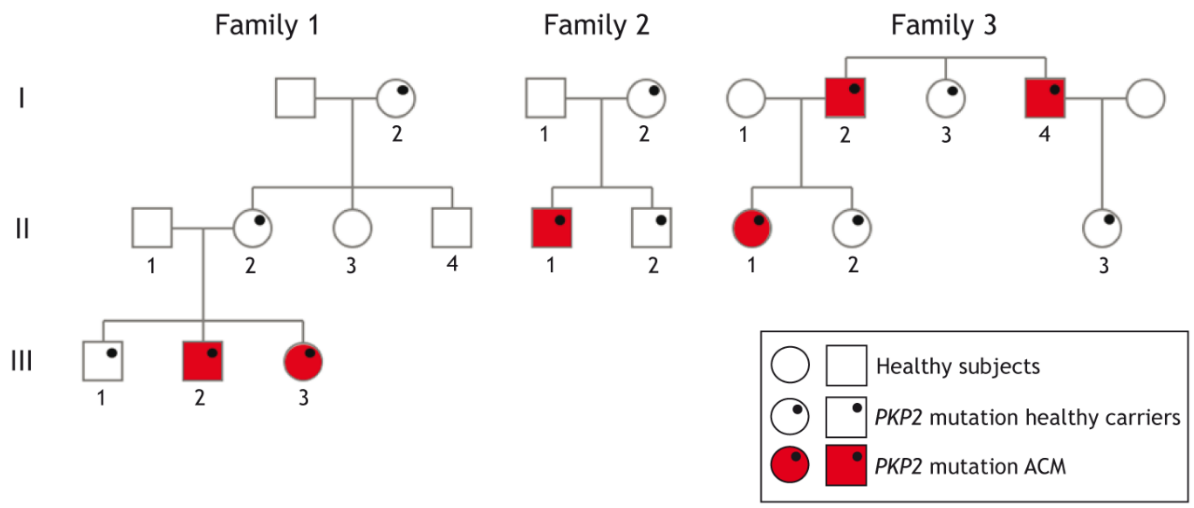

Appendix Figure S1. Families in which investigation of oxidative stress and dyslipidemia as genetically determined secondary traits was conducted. The pedigrees of three genotyped ACM families are represented. DNA of all numbered individuals underwent exome sequencing. Full red blocks represent subjects with ACM, black circles represent main causative mutations.

Dataset EV1. List of genes and variants linked to oxidative stress and dyslipidemia analyzed

Sheet 1: List of genes linked to oxidative stress and dyslipidemia analyzed for genetic variants; sheet 2: list of variants segregating with the phenotype in an analysis including all three families (pedigrees shown); sheet 3: list of variants segregating with the phenotype in the analysis of family 1 (pedigree shown); sheet 4: list of variants segregating with the phenotype in the analysis of family 2 (pedigree shown); sheet 5: list of variants segregating with the phenotype in the analysis of family 3 (pedigree shown).

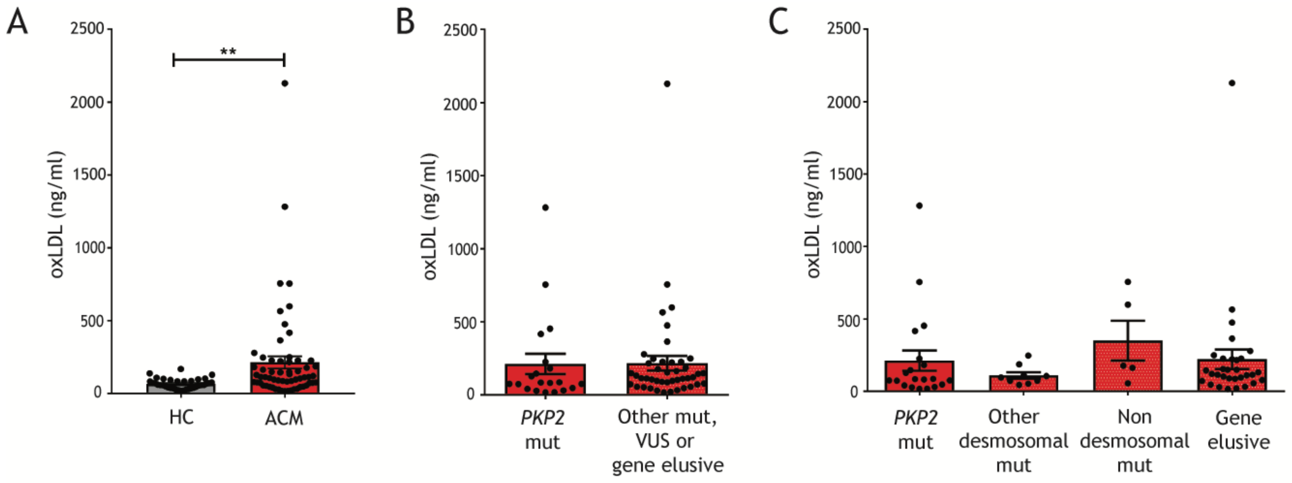

Appendix Figure S2. ACM patient genetics do not influence oxLDL plasma levels.

A) oxLDL plasma concentration in the whole cohort of ACM patients and HC (n=65 vs. n=36; Mann-Whitney test). B) oxLDL plasma concentration in ACM patients carriers of a *PKP2* mutation and ACM patients carriers of other desmosomal or non desmosomal mutations, or gene elusive (n=20 vs. n=45). C) oxLDL plasma concentration in ACM patients carriers of a *PKP2* mutation, ACM patients carriers of other desmosomal mutations, ACM patients carriers of non desmosomal mutations, or gene elusive ACM patients (n=20 vs. n=9 vs. n=5 vs. n=31). **$p<0.01$

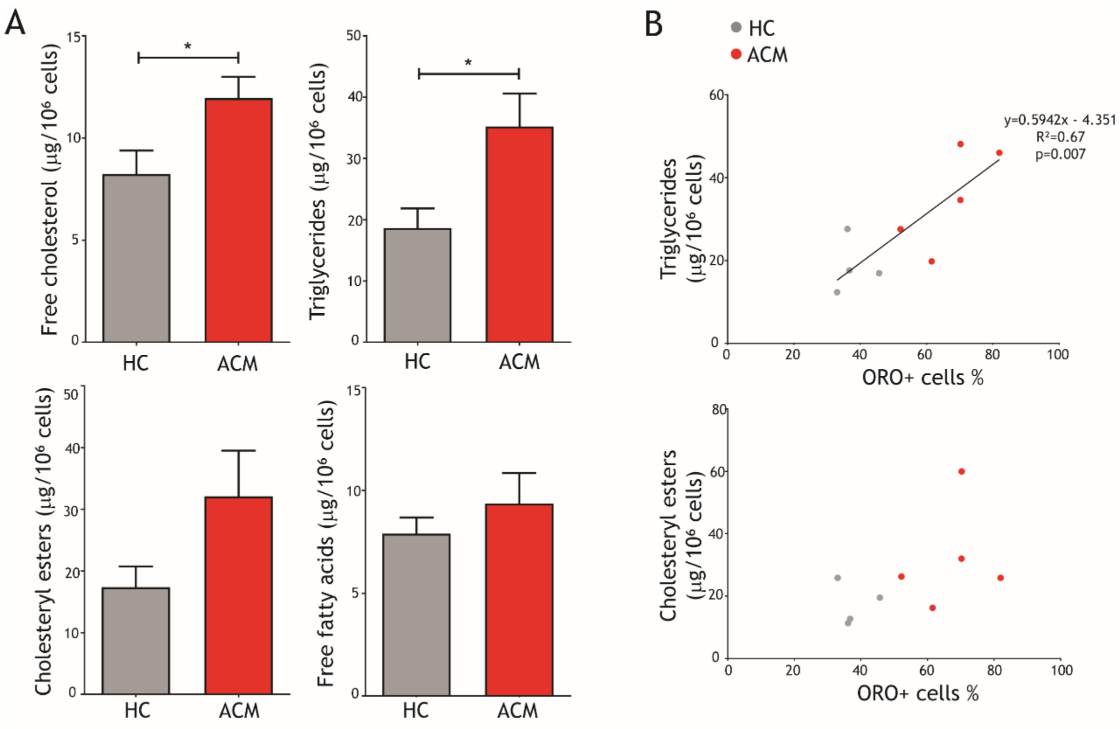

Appendix Figure S3. Comparison of the mass of different lipid classes accumulated by HC and ACM C-MSC during adipogenic differentiation.

A) Mass of free cholesterol, triglycerides, cholesteryl esters and free fatty acids accumulated by HC (n=4) and ACM (n=5; Two-tailed Student's *t*-test) C-MSC after 7 days of adipogenic differentiation. The masses are normalized on 10^6 cells. * $p<0.05$. **B**) Upper panel: correlation between triglyceride mass and percentage of ORO positive cells. Regression line, its equation, R^2 and p value are shown (X-Y correlation). Lower panel: dot plot of cholesteryl ester mass and percentage of ORO positive cells.

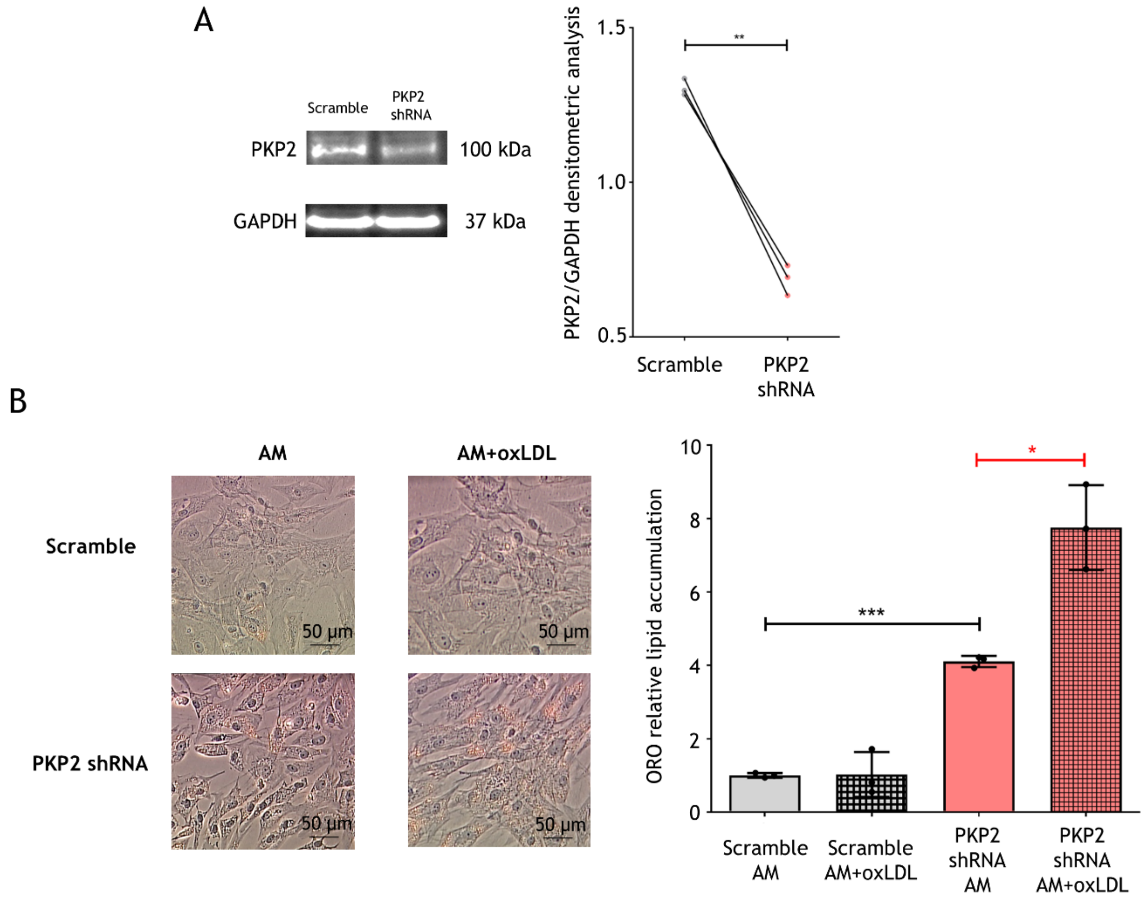

Appendix Figure S4. *PKP2* silencing in HC C-MSC demonstrated that the susceptibility to oxLDL is dependent on genetic causes.

A) Left panel: Western Blot of PKP2 and GAPDH protein expression of healthy control (HC) cardiac mesenchymal stromal cell (C-MSC) protein extracts treated with shRNA scrambled control or PKP2 shRNA and cultured in growth medium (GM). Right panel: densitometric analysis normalized on the housekeeping protein GAPDH (n = 3 each). ** $p < 0.01$ (Two-tailed Student's t-test).

B) Oil Red O (ORO) staining quantification of HC C-MSC treated with shRNA scrambled control or PKP2 shRNA and cultured in adipogenic medium (AM) with or without 150 µg/ml oxLDL for 72 hours (n = 3 each). * $p < 0.05$, *** $p < 0.001$ (One-Way Anova).

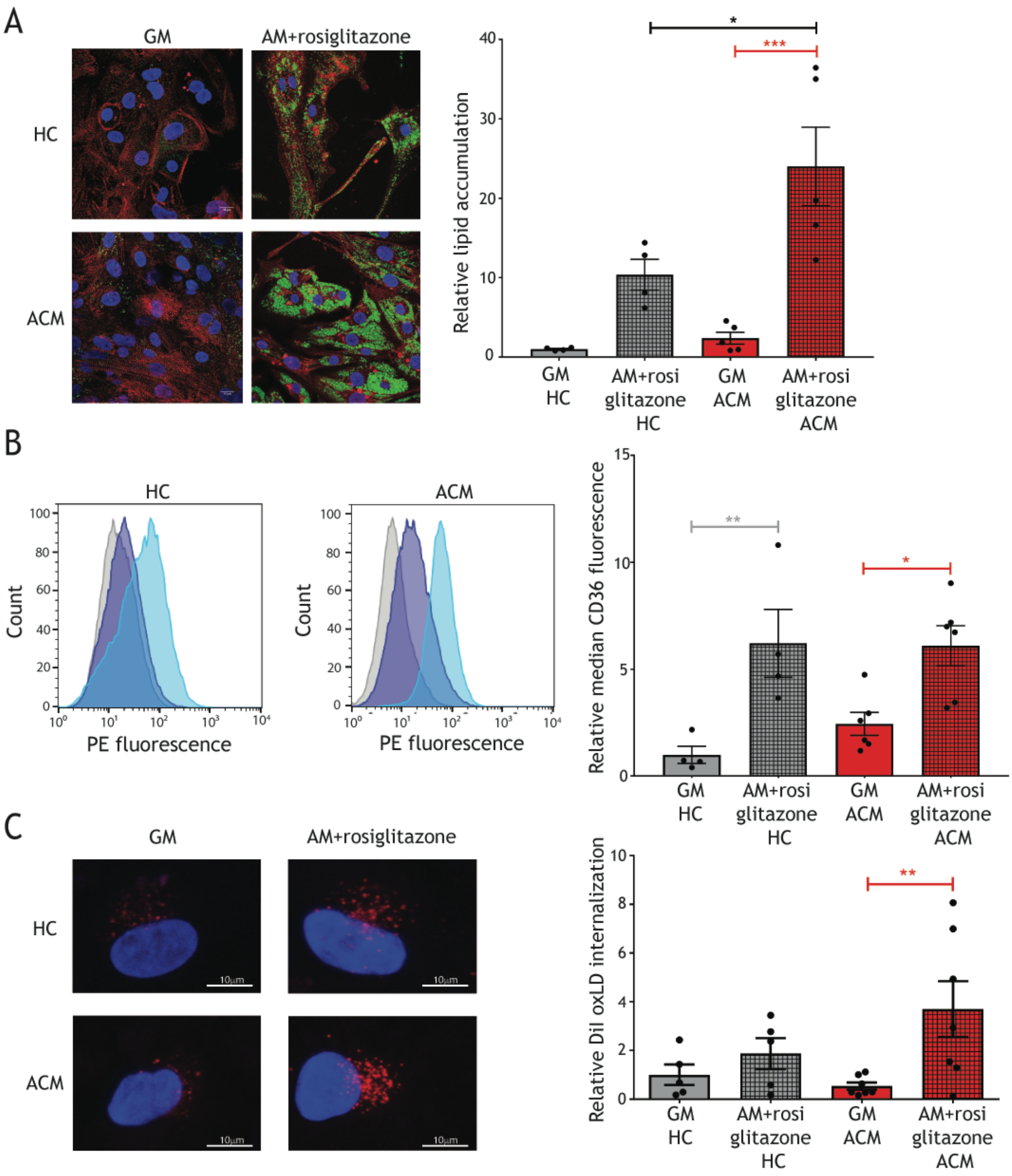

Appendix Figure S5. Lipid accumulation and CD36 levels increase in hiPSC-CM upon PPARγ agonism.

A) Representative images of lipid accumulation, detected with BODIPY (green), and cardiomyocyte marker αSARC (red) co-localization in ACM and HC hiPSC-CM cultured in GM or AM added with 5µM rosiglitazone. Nuclei are stained with DAPI. On the right, the relative quantification of lipid accumulation is shown. Relative lipid accumulation is presented as normalized to GM HC (Two-Way Anova). **B**) Left panels: exemplificative FACS analysis of the PE fluorescence of unstained HC and ACM hiPSC-CM (autofluorescence; gray), in GM stained with CD36 antibodies (purple), and in AM added with 5µM rosiglitazone stained with CD36 antibodies (light blue). Right panel: quantification of the median CD36

fluorescence of HC and ACM hiPSC-CM both in GM and in AM added with 5µM rosiglitazone. Relative median CD36 fluorescence is presented as normalized to GM HC (Two-Way Anova). **C**) Left panels: exemplificative figures of DI oxLDL internalization in HC and ACM cells, both in GM and in AM added with 5µM rosiglitazone. Right panel: quantification of the relative mean DI fluorescence normalized on nuclei number for each sample (n=5 HC n=7 ACM; Two-Way Anova). * $p<0.05$, ** $p<0.01$, *** $p<0.001$.

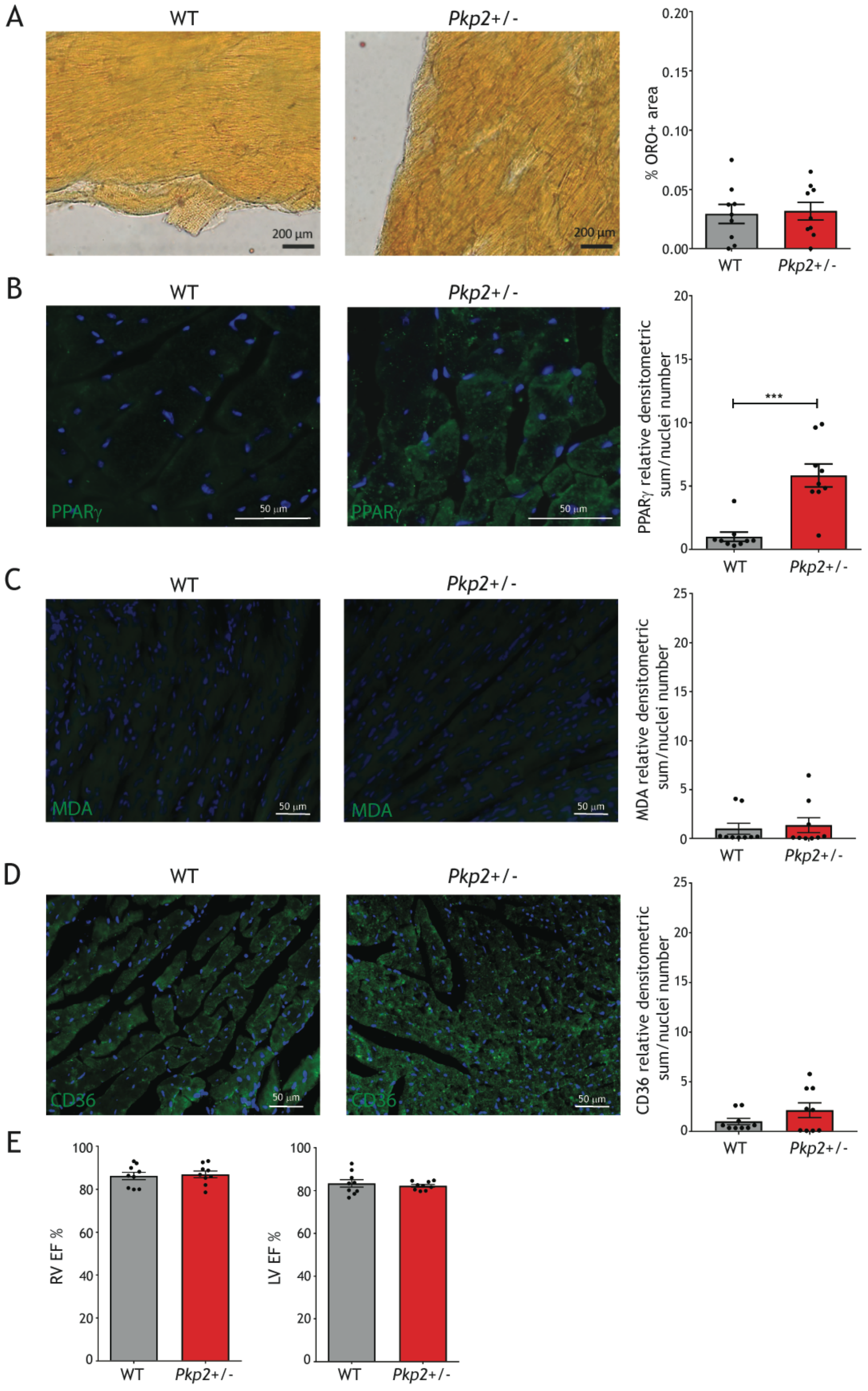

Appendix Figure S6. Characterization of *Pkp2*+/- model in CD.

A) Left panels: representative images of ventricular sections of WT and *Pkp2*+/- mice, stained with ORO. Right panel: quantification of the percentage of ORO positive cardiac tissue area (n=9 each; Two-tailed Student's *t*-test). **B**) Left panels: representative images of PPARγ (green) immunostaining on ventricular tissue

sections of WT and *Pkp2*+/- mice. Nuclei are counterstained with Hoechst 33342 (blue). Right panel: image quantification (n=9 each; Two-tailed Student's *t*-test). **C**) Left panels: representative images of MDA (green) immunostaining on ventricular tissue sections of WT and *Pkp2+/-* mice. Nuclei are counterstained with Hoechst 33342 (blue). Right panel: image quantification (n=9 each; Two-tailed Student's *t*-test). **D**) Left panels: representative images of CD36 immunostaining (green) on ventricular tissue sections of WT and *Pkp2*+/- mice. Nuclei are counterstained with Hoechst 33342 (blue). Right panel: image quantification (n=9 each; Two-tailed Student's *t*-test). **E**) Echocardiographic parameters of WT and *Pkp2*+/- mice. The percentages of RV EF % and LV EF % are shown (n=9 each; Two-tailed Student's *t*-test). * $p<0.05$.

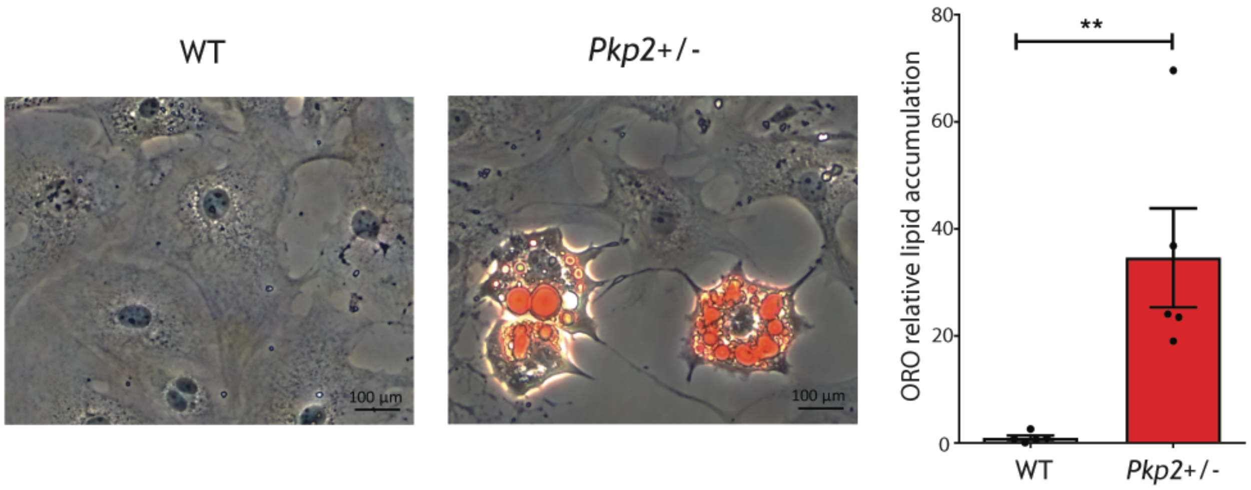

Appendix Figure S7. *Pkp2*+/- C-MSC are prone to adipogenic differentiation *in vitro*.

Left panels: representative images of ORO stained C-MSC isolated from WT and *Pkp2*+/- mice and cultured in adipogenic medium for 5 days. Right panel: quantification of C-MSC lipid accumulation (n=5 each; Two-tailed Student's *t*-test). ** p<0.01.

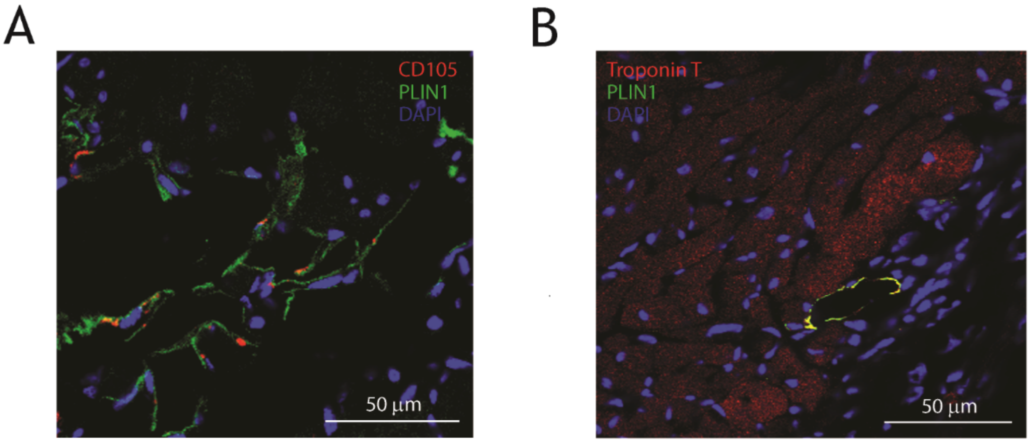

Appendix Figure S8. C-MSC undergo adipogenic differentiation in murine hearts with HFD.

A) Representative image of adipose accumulation, detected with PLIN1 antibody (green), and mesenchymal marker CD105 (red) co-localization in *Pkp2*+/- heart section after HFD. Nuclei are stained with Hoechst 33258. **B**) Representative image of immunofluorescence staining of *Pkp2*+/- heart section after HFD with PLIN1 antibody (green) for fat droplets membranes and Troponin T antibody (red) for cardiomyocytes. Nuclei are stained with Hoechst 33258. No co-localization was detected. HFD: high fat diet; Pkp2+/-: plakophilin2 heterozygous knock-out; PLIN1: Perilipin1.

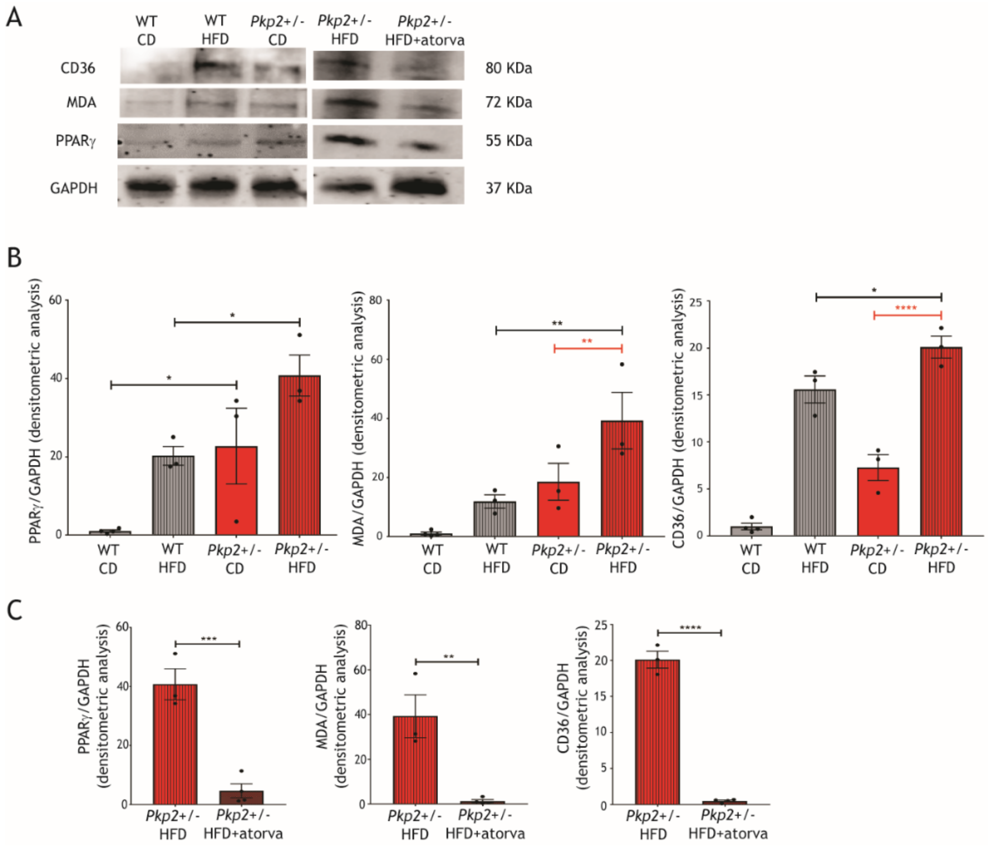

Appendix Figure S9. Validation by Western Blot of the quantification of the immunofluorescence analyses performed on mouse hearts.

A) Representative images of Western Blot analysis of proteins extracted from CD-, HFD-, and HFD+atorva-fed WT and *Pkp2*+/- total heart tissue hybridized with anti-PPARγ, anti-MDA, and anti-CD36 and antibodies. Immunostaining of the housekeeping GAPDH is shown for normalization. **B)** Densitometric analysis of cardiac PPARγ, MDA, and CD36 levels, normalized on GAPDH, of CD- and HFD-fed WT and *Pkp2*+/- mice (n=3; Two-Way Anova). **C)** Densitometric analysis of cardiac PPARγ, MDA, and CD36 levels, normalized on GAPDH, of HFD- and HFD+atorvastatin-fed *Pkp2*+/- mice (n=3; Two-tailed Student's *t*-test). * $p<0.05$ ** $p<0.01$ *** $p<0.001$

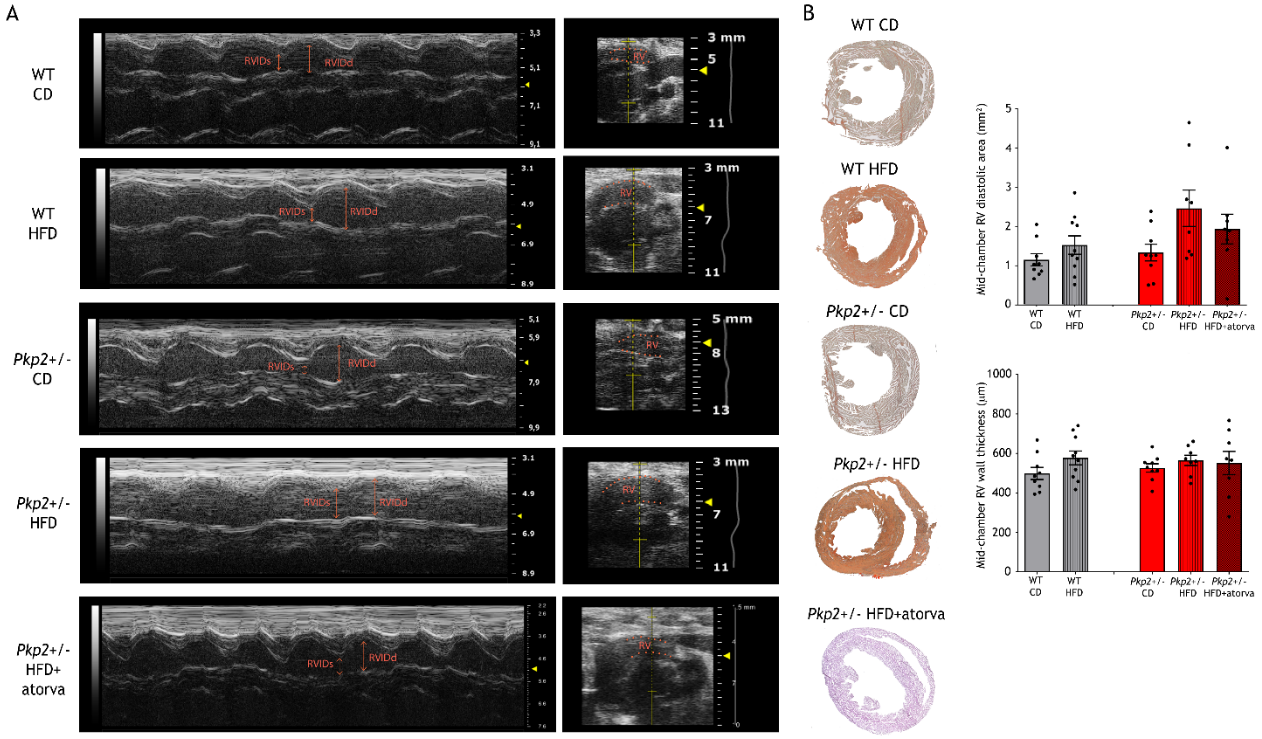

Appendix Figure S10. Echocardiographic and morphometric parameters of WT and *Pkp2*+/- mice before and after HFD and HFD+atorva.

A) Representative two-dimensional echocardiograms (M-mode, left panels; B-mode, right panels) of WT and *Pkp2*+/- mice in CD (upper panels) and after 3 months of HFD and HFD+atorva (lower panels). The RVIDs and RVIDd and the RV are depicted. **B**) Left panels: Oil Red O–stained representative sections from the cardiac mid-chamber of WT and *Pkp2*+/- mice in CD and after HFD and HFD+atorva. Right panels: mid-chamber RV diastolic area and mid-chamber RV wall thickness (n=9 each for CD; n=10 each HFD; n=9 for *Pkp2*+/- HFD+atorva).

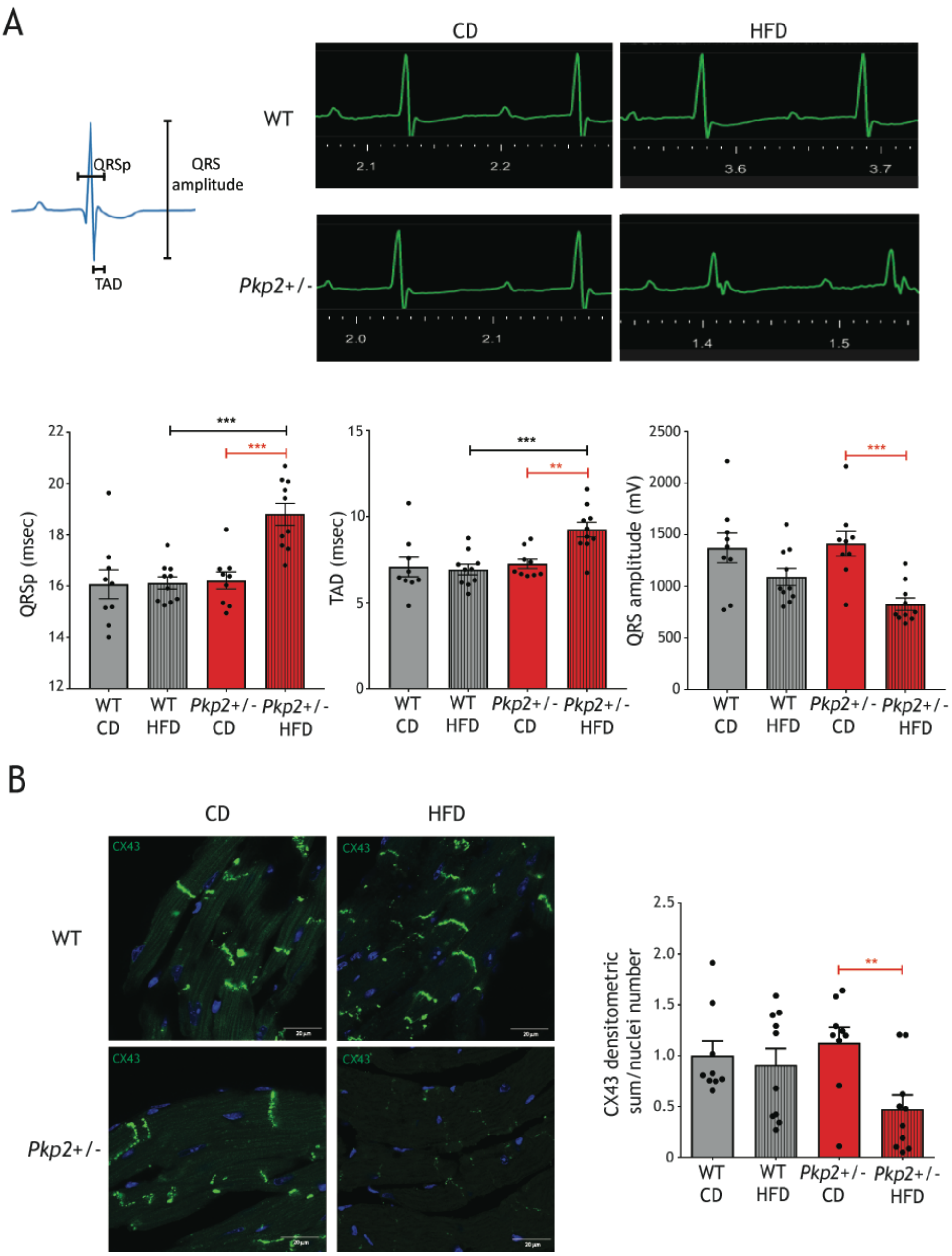

Appendix Figure S11. HFD administration leads to electrical dysfunction in *Pkp2+/-* mice

A) Representative surface electrocardiograms (ECG; upper right panels) of WT and *Pkp2*+/- mice in CD and after 3 months of HFD. The QRSp and TAD intervals, as well as QRS amplitude were measured as described on the upper left panel. Lower panels: quantification of the QRSp and TAD intervals, and QRS amplitude (n=9 each for CD; n=10 each HFD; Two-Way Anova). **B**) Left panels: representative images of CX43 (green) immunostaining on ventricular tissue sections of CD- and HFD-fed WT and *Pkp2*+/- mice. Nuclei are stained with Hoechst 33258. Right panels: image quantification (n=9 each for CD; n=10 each HFD; Two-Way Anova). ** $p<0.01$ *** $p<0.001$

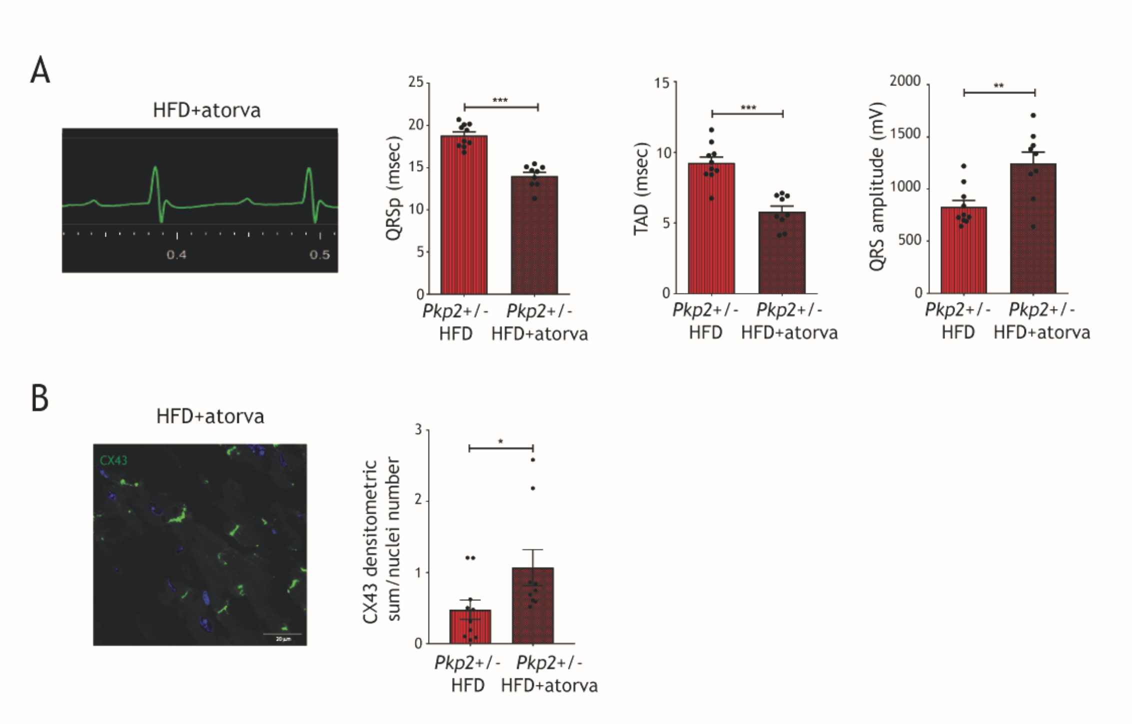

Appendix Figure S12. Atorvastatin treatment prevents electrical dysfunction in *Pkp2+/-* mice.

A) Representative surface electrocardiograms (ECG; left panel) of *Pkp2*+/- mice after 3 months of HFD+atorva. Right panels: quantification of the QRSp and TAD intervals, and QRS amplitude compared to that in *Pkp2+/-* HFD (n=10 for HFD; n=9 HFD+atorva; Two-Way Anova). **B**) Left panel: representative image of CX43 (green) immunostaining on ventricular tissue sections of HFD+atorva-fed *Pkp2*+/- mice. Nuclei are stained with Hoechst 33258. Right panel: image quantification compared to that in *Pkp2+/-* HFD (n=10 for HFD; n=9 HFD+atorva; Two-Way Anova). * $p<0.05$ ** $p<0.01$ *** $p<0.001$

Supplemental Tables:

Appendix Table S1. Clinical parameters of the individuals enrolled for the plasma analysis.

	HC	ACM	p value
**n**	36	36	-
**Age (years)**	44±2	46±2	p=0.55
**Male gender (n)**	30	30	p=1.00
**Sport (>3 times/week) (n)**	13	12	p>0.99
**Smoking (n)**	8	9	p>0.99
**Family history of cardiovascular events (n)**	5	7	p=0.75
**Hypertension (n)**	7	12	p=0.28
**Obesity (n)**	0	1	p>0.99
**Diabetes (n)**	0	1	p>0.99
**Total cholesterol (mg/dl)**	188.5(166.8-214.8)	196.5(166.5-225.3)	p=0.52
**LDL cholesterol (mg/dl)**	114.0(102.8-138.7)	121.5(89.25-148.8)	p=0.74
**OxLDL (ng/ml)**	59.23(38.55-85.6)	92.31(42.62-201.2)	* p=0.015
**13HODE (ng/ml)**	24.8(16.7-34.4)	43.4(20.2-63.0)	* p=0.03
**Mutation in known ACM genes (n; %)**	na	19 on 34 genotyped; 55.9%	-

Continuous variables are expressed as median(IQR). The indicated p value is the result of Fisher's exact test for discrete variables and Mann-Whitney test for continuous variables. * $p<0.05$.

13HODE: 13-hydroxy-octadecadienoic acid; ACM: Arrhythmogenic Cardiomyopathy; HC: healthy controls; LDL: low density lipoproteins; n: number of subjects; na: not available; oxLDL: oxidized low density lipoproteins.

Appendix Table S2. Characteristics of ACM patients with a known ACM associated mutations and their family members, carriers of the same mutation but clinically not affected by ACM, enrolled for the plasma analysis.

	Mutation carrier NON ACM	Mutation carrier ACM	p value
**n**	9	7	-
**Age (years)**	49±6	45±6	p=0.67
**Male gender (n; %)**	4; 44.4%	5; 71.4%	p=0.36
**Obesity (n)**	0	0	p=1.00
**OxLDL (ng/ml)**	49.19(30.87-89.44)	303.3(71.23-754.5)	* p=0.03
**Mutation in known ACM genes (n; %)**	9; 100%	7; 100%	-

Continuous variables are expressed as median(IQR). The indicated p value is the result of Fisher's exact test for discrete variables and Mann-Whitney test for continuous variables. * $p<0.05$.

ACM: Arrhythmogenic Cardiomyopathy; n: number of subjects; na: not available; oxLDL: oxidized low density lipoproteins.

Appendix Table S3. Genotypes of the individuals enrolled for the plasma analysis. na: not available

Gene	Type of variant	Number of patients	% of the genotyped cohort
PKP2	pathogenic	11	32.4%
	unknown	1	2.9%
DSG2	pathogenic	0	-
	unknown	2	5.9%
DSC2	pathogenic	0	-
	unknown	1	2.9%
JUP	pathogenic	0	-
	unknown	1	2.9%
TMEM43	pathogenic	0	-
	unknown	1	2.9%
DES	pathogenic	1	2.9%
	unknown	1	2.9%
Gene elusive	-	15	44.1%
na	-	2	-
**TOTAL**		**36 of which 34 genotyped**	**100%**

Appendix Table S4A. Variant in genes of the oxidative stress and dyslipidemia panel (Dataset EV1) in all the three analyzed families.

Family	Variant	Allele frequency in the general population	Gene	Pathway	Classification	ACMG/AMP classification	Allele frequency in healthy carriers	Allele frequency in affected carriers
ALL	rs77176546	0.22	*MGST3*	Glutathione-dependent peroxidase activity	Oxidative stress	na	0.3125	0.6667

Appendix Table S4B. Variant in genes of the oxidative stress and dyslipidemia panel (Dataset EV1) found separately in each family.

Family	Variant	Allele frequency in the general population	Gene	Pathway	Classification	ACMG/AMP classification
1	rs11087654	0.004691	*PRNP*	ROS detoxification	Oxidative stress	Benign
1	rs17248882	0.002529	*LDLR*	LDL internalization	Dyslipidemia	Uncertain Significance
2	na	0.0008196	*VIMP*	Anti-oxidant function	Oxidative stress	na
2	rs199922141	na	*CYP2E1*	ROS generator	Oxidative stress	Uncertain Significance
2	na	na	*TXNRD1*	Redox homeostasis	Oxidative stress	na
2	rs142623210	0.001156	*NFKB2*	Increase expression of antioxidant proteins	Oxidative stress	Benign
2	rs5742620	0.023	*IGF1*	Mitochondrial protection and antioxidant function	Oxidative stress	Benign
2	rs55676195	6.619e-05	*TTN*	Reduced cardiac contractility when oxidized	Oxidative stress	Likely Benign
2	rs55886356	0.006771	*TTN*	Reduced cardiac contractility when oxidized	Oxidative stress	Benign
2	rs34070843	0.018	*TTN*	Reduced cardiac contractility when oxidized	Oxidative stress	Benign
2	rs115744476	0.005594	*TTN*	Reduced cardiac contractility when oxidized	Oxidative stress	Benign
2	rs185767460	na	*TTN*	Reduced cardiac contractility when oxidized	Oxidative stress	Likely Benign
3	rs201428532	0.000313	*TRPM2*	Activated by oxidative stress	Oxidative stress	Uncertain Significance

LDL: low density lipoproteins; na: not available; redox: oxidation-reduction; ROS: reactive oxygen species.

Appendix Table S5. Genotypes of the whole cohort of ACM patients. na: not available.

Gene	Type of variant	Number of patients	% of the genotyped cohort
PKP2	pathogenic	17	26.1%
	unknown	3	4.6%
DSG2	pathogenic	0	-
	unknown	3	4.6%
DSP	pathogenic	1	1.5%
	unknown	0	-
DSC2	pathogenic	2	3.1%
	unknown	2	3.1%
JUP	pathogenic	0	-
	unknown	1	1.5%
TMEM43	pathogenic	0	-
	unknown	2	3.1%
DES	pathogenic	1	1.5%
	unknown	2	3.1%
Gene elusive	-	31	47.7%
na	-	2	-
**TOTAL**	**-**	67 of which 65 genotyped	100%

Appendix Table S6. List of the C-MSC obtained from ACM and HC individuals used for the *in vitro* experiments (dependent on availability and culture passage number).

13H: 13-hydroxy-octadecadienoic acid; ACM: Arrhythmogenic Cardiomyopathy; DCF analysis: 2′,7′-Dichlorofluorescein diacetate analysis; GSH/GSSG ratio: reduced glutathione/oxidized glutathione ratio; MDA IF: Malondialdehyde immunofluorescence; NAC: N-acetylcysteine; NR: Nile Red; oxLDL intern.: oxLDL internalization; WB: western blot. For ACM patients only, genotype is indicated. Only pathogenic, likely pathogenic or unknown variants are reported.

Sample name	Sex	Age	Genotype	DCF analysis	MDA IF	GSH/ GSSG ratio	WB in basal conditions	CD36/ NR FACS analysis	OxLDL intern.	OxLDL treatment	13H/ NAC treatment	CD36 siRNA	Lipid analysis
**ACM1**	M	52	*PKP2*: c.2013delC; p.K672RfsX11	X			X	X					X
**ACM2**	M	42	*PKP2*: c.1643delG; p.V548fsX562	X			X	X					X
**ACM3**	M	40	*PKP2*: c.1881delC; p.K628RfsX12	X		X	X	X		X	X		X
**ACM4**	M	51	negative	X		X	X	X			X		X
**ACM5**	M	57	negative	X		X	X					X	X
**ACM6**	M	43	*DSP*: c.6850C>T; p.R2284X				X				X		
**ACM7**	F	39	*PKP2*: c.2013delC; p.K672RfsX11				X				X		
**ACM8**	M	64	negative				X				X		
**ACM9**	M	45	*DSC2:* c.416C>T; p.P139L				X			X			
**ACM10**	M	46	*DSG2*: c.1003A>G; p.T335A			X	X			X	X	X	
**ACM11**	M	47	negative			X	X			X	X	X	
**ACM12**	M	30	*TMEM43:* c.718C>T; p.R240C			X	X			X	X	X	
**ACM13**	F	42	negative		X	X	X				X		
**ACM14**	M	41	*PKP2:* c.548G>A; p.P119L		X		X			X	X	X	
**ACM15**	F	24	*DSG2:* c.1003A>G; p.T335A		X		X			X	X		
**ACM16**	F	52	*JUP*: c.1359G>T; p.E453D		X		X			X	X		
**ACM17**	M	49	negative			X	X		X	X			
**ACM18**	M	52	negative				X		X	X	X	X	
**ACM19**	M	28	negative				X		X	X		X	

Sample name	Sex	Age	DCF analysis	MDA IF	GSH/ GSSG analysis	WB in basal conditions	CD36/ NR FACS analysis	OxLDL intern.	OxLDL treatment	13H/ NAC treatment	CD36 siRNA	Lipid analysis
**HC1**	M	51	X			X	X			X		X
**HC2**	M	48	X		X	X	X			X		X
**HC3**	M	44	X			X	X			X		X
**HC4**	M	56	X			X	X		X	X		X
**HC5**	M	40	X	X	X	X	X					X
**HC6**	F	25		X		X				X		
**HC7**	M	57		X	X	X						
**HC8**	F	50		X		X			X			
**HC9**	F	35		X	X	X			X	X		
**HC10**	M	41				X			X	X		
**HC11**	M	55			X	X			X	X		
**HC12**	M	17				X			X	X		
**HC13**	M	57			X	X						
**HC14**	F	58				X						
**HC15**	M	21			X	X		X				
**HC16**	M	49			X	X		X	X			
**HC17**	M	64				X			X	X		
**HC18**	F	48				X			X	X		
**HC19**	M	44				X		X	X	X		
**HC20**	M	28				X			X	X		

Appendix Table S7. Antibody list.

	Protein	Antibody	Host	Company	Dilution
**Anti-human**	MDA	ab6463	Rabbit	Abcam	IF 1:2500
	CD36	610882	Mouse	BD	IF 1:200- WB 1:1000
	CD36	A15724 (clone CB38 – NL07)	Mouse	Life Technologies	FACS on C-MSC 1:50
	CD36	A15777 (clone TR9)	Mouse	Life Technologies	FACS on hiPSC-CM 1:100
	GAPDH	sc-25778	Rabbit	Santa Cruz	WB 1:1000
	PPARγ	sc-7273 (clone E-8)	Mouse	Santa Cruz	WB 1:60
	αSARC	A7732	Mouse	Sigma-Aldrich	IF 1:250
**Anti-mouse**	MDA	ab6463	Rabbit	Abcam	IF 1:2500- WB 1:1000
	CD36	RA25035	Rabbit	Neuromics	IF 1:200- WB 1:1000
	PPARγ	PA3-821A	Rabbit	Life Technologies	IF 1:200- WB 1:1000
	PLIN1	BP5015	Guinea Pig	OriGene	IF 1:100
	CD105	BAF1320	Goat	R&D System	IF 1:40
	Troponin T	ab92546	Rabbit	Abcam	IF 1:100
	Connexin 43	ab11370	Rabbit	Abcam	IF 1:400
**Secondary antibodies**	Anti-rabbit IgG 488	A11034	Goat	Life Technologies	IF 1:200
	Anti-mouse IgG 488	A11001	Goat	Life Technologies	IF 1:200
	Anti-mouse IgG HRP	GENA9310	Sheep	GE Healthcare	WB 1:1000
	Anti-rabbit IgG HRP	GENA9340	Donkey	GE Healthcare	WB 1:1000
	Anti-mouse IgG 555	A28180	Goat	Life Technologies	IF 1:1000
	Anti-guinea pig 488	sc-2441	Goat	Santa Cruz	IF 1:200
	Anti-streptavidin 594	S32356	-	Life Technologies	IF 1:200
	Anti-rabbit 546	A11010	Goat	Life Technologies	IF 1:200

Appendix Table S8. P-Value list.

Figure	p-value
Figure 1A	p=0.015
Figure 1B	p=0.02
Figure 1C (PKP2 mut vs. other desmosomal mut)	p>0.9999
Figure 1C (PKP2 mut vs. non desmosomal mut)	p>0.9999
Figure 1C (PKP2 mut vs. gene elusive)	p>0.9999
Figure 1C (other desmosomal mut vs. non desmosomal mut)	p>0.9999
Figure 1D	p=0.015
Figure 1E	p=0.0007
Figure 2B	p=0.04
Figure 2C	p=0.04
Figure 2D	p=0.02
Figure 2E	p=0.01
Figure 2F	p=0.04
Figure 3A	p=0.049
Figure 3B	p=0.017
Figure 3C	p=0.942
Figure 3D (PPARγ)	p=0.002
Figure 3D (CD36)	p=0.02
Figure 3E (HC)	p=0.03
Figure 3E (ACM)	p=0.008
Figure 3F (ACM GM vs. AM)	p=0.002
Figure 3F (HC GM vs. AM)	p=0.059
Figure 3F (ACM AM vs. HC AM)	p=0.01
Figure 3F (ACM GM vs. HC GM)	p=0.371
Figure 4A (ORO ACM AM vs. AM+oxLDL)	p=0.01
Figure 4A (ORO HC AM vs. AM+oxLDL)	p>0.9999
Figure 4A (ORO ACM AM vs. HC AM)	p=0.028
Figure 4A (ORO ACM AM+oxLDL vs. HC AM+oxLDL)	p<0.0001
Figure 4A (PPARγ ACM AM vs. AM+oxLDL)	p=0.01
Figure 4A (PPARγ HC AM vs. AM+oxLDL)	p>0.9999
Figure 4A (PPARγ ACM AM vs. HC AM)	p=0.193
Figure 4A (PPARγ ACM AM+oxLDL vs. HC AM+oxLDL)	p=0.002
Figure 4A (CD36 ACM AM vs. AM+oxLDL)	p=0.01
Figure 4A (CD36 HC AM vs. AM+oxLDL)	p>0.9999
Figure 4A (CD36 ACM AM vs. HC AM)	p=0.332
Figure 4A (CD36 ACM AM+oxLDL vs. HC AM+oxLDL)	p=0.0007
Figure 4B (ACM AM vs. AM+13HODE)	p=0.048
Figure 4B (ACM AM+13HODE vs. AM+NAC)	p<0.0001
Figure 4B (ACM AM+13HODE vs. AM+13HODE+NAC)	p=0.02
Figure 4B (ACM AM vs. AM+NAC)	p=0.03
Figure 4B (ACM AM vs. AM+13HODE+NAC)	p>0.9999
Figure 4B (ACM AM+NAC vs. AM+13HODE+NAC)	p>0.9999
Figure 4B (HC AM vs. AM+13HODE)	p=0.946
Figure 4B (HC AM+13HODE vs. AM+NAC)	p>0.9999
Figure 4B (HC AM+13HODE vs. AM+13HODE+NAC)	p>0.9999
Figure 4B (HC AM vs. AM+NAC)	p>0.9999
Figure 4B (HC AM vs. AM+13HODE+NAC)	p=0.380
Figure 4B (HC AM+NAC vs. AM+13HODE+NAC)	p>0.9999
Figure 4B (HC AM vs. ACM AM)	p=0.012
Figure 4B (HC AM+13HODE vs. ACM AM+13HODE)	p=0.001
Figure 4B (HC AM+NAC vs. ACM AM+NAC)	p>0.9999
Figure 4B (HC AM+13HODE+NAC vs. ACM AM+13HODE+NAC)	p=0.203
Figure 4C (PPARγ ACM AM vs. AM+13HODE)	p=0.047
Figure 4C (PPARγ ACM AM+13HODE vs. AM+NAC)	p<0.0001
Figure 4C (PPARγ ACM AM+13HODE vs. AM+13HODE+NAC)	p=0.0001
Figure 4C (PPARγ ACM AM vs. AM+NAC)	p=0.007
Figure 4C (PPARγ ACM AM vs. AM+13HODE+NAC)	p=0.314

Figure 4C (PPARγ ACM AM+NAC vs. AM+13HODE+NAC)	p=0.841
Figure 4C (PPARγ HC AM vs. AM+13HODE)	p>0.9999
Figure 4C (PPARγ HC AM+13HODE vs. AM+NAC)	p>0.9999
Figure 4C (PPARγ HC AM+13HODE vs. AM+13HODE+NAC)	p>0.9999
Figure 4C (PPARγ HC AM vs. AM+NAC)	p>0.9999
Figure 4C (PPARγ HC AM vs. AM+13HODE+NAC)	p>0.9999
Figure 4C (PPARγ HC AM+NAC vs. AM+13HODE+NAC)	p>0.9999
Figure 4C (PPARγ HC AM vs. ACM AM)	p=0.005
Figure 4C (PPARγ HC AM+13HODE vs. ACM AM+13HODE)	p<0.0001
Figure 4C (PPARγ HC AM+NAC vs. ACM AM+NAC)	p=0.989
Figure 4C (PPARγ HC AM+13HODE+NAC vs. ACM AM+13HODE+NAC)	p=0.391
Figure 4C (CD36 ACM AM vs. AM+13HODE)	p=0.008
Figure 4C (CD36 ACM AM+13HODE vs. AM+NAC)	p<0.0001
Figure 4C (CD36 ACM AM+13HODE vs. AM+13HODE+NAC)	p<0.0001
Figure 4C (CD36 ACM AM vs. AM+13HODE+NAC)	p<0.0001
Figure 4C (CD36 ACM AM+NAC vs. AM+13HODE+NAC)	p>0.9999
Figure 4C (CD36 HC AM vs. AM+13HODE)	p>0.9999
Figure 4C (CD36 HC AM+13HODE vs. AM+NAC)	p>0.9999
Figure 4C (CD36 HC AM+13HODE vs. AM+13HODE+NAC)	p>0.9999
Figure 4C (CD36 HC AM vs. AM+NAC)	p>0.9999
Figure 4C (CD36 HC AM vs. AM+13HODE+NAC)	p>0.9999
Figure 4C (CD36 HC AM+NAC vs. AM+13HODE+NAC)	p>0.9999
Figure 4C (CD36 HC AM vs. ACM AM)	p=0.985
Figure 4C (CD36 HC AM+13HODE vs. ACM AM+13HODE)	p<0.0001
Figure 4C (CD36 HC AM+NAC vs. ACM AM+NAC)	p>0.9999
Figure 4C (CD36 HC AM+13HODE+NAC vs. ACM AM+13HODE+NAC)	p>0.9999
Figure 5A (CD36)	p=0.014
Figure 5A (PPARγ)	p=0.05
Figure 5B (ORO)	p=0.003
Figure 5C (CD36/PPARγ correlation)	p=0.0002
Figure 5C (CD36/ORO correlation)	p=0.07
Figure 5D	p=0.04
Figure 5E	p=0.01
Figure 5F	p=0.04
Figure 6A	p<0.0001
Figure 6B	p=0.049
Figure 6C (WT HFD vs. *Pkp2*+/- HFD)	p<0.0001
Figure 6C (WT CD vs. *Pkp2*+/- CD)	p>0.9999
Figure 6C (WT CD vs. WT HFD)	p>0.9999
Figure 6C (*Pkp2*+/- CD vs. *Pkp2*+/- HFD)	p=0.0006
Figure 6D (WT HFD vs. *Pkp2*+/- HFD)	p=0.0001
Figure 6D (WT CD vs. *Pkp2*+/- CD)	p=0.011
Figure 6D (WT CD vs. WT HFD)	p=0.876
Figure 6D (*Pkp2*+/- CD vs. *Pkp2*+/- HFD)	p=0.063
Figure 6E (WT HFD vs. Pkp2+/- HFD)	p=0.0003
Figure 6E (WT CD vs. *Pkp2*+/- CD)	p>0.9999
Figure 6E (WT CD vs. WT HFD)	p>0.9999
Figure 6E (*Pkp2*+/- CD vs. *Pkp2*+/- HFD)	p=0.0461
Figure 6F (WT HFD vs. Pkp2+/- HFD)	p=0.04
Figure 6F (WT CD vs. *Pkp2*+/- CD)	p=0.918
Figure 6F (WT CD vs. WT HFD)	p=0.378
Figure 6F (*Pkp2*+/- CD vs. *Pkp2*+/- HFD)	p=0.004
Figure 6G (RV EF)	p=0.02
Figure 6G (RV FS)	p=0.02
Figure 6G (RVID systole)	p=0.03
Figure 6G (RVID diastole)	p>0.9999
Figure 7A	p<0.001
Figure 7B	p=0.009
Figure 7C	p=0.001

Figure 7D	p<0.0001
Figure 7E	p=0.0003
Figure 7F	p=0.001
Figure 7G (RV EF)	p=0.04
Figure 7G (RV FS)	p=0.04
Figure 7G (RVID systole)	p=0.029
Figure 7G (RVID diastole)	p=0.266
Appendix Figure S2A	p=0.009
Appendix Figure S2B	p=0.471
Appendix Figure S2C (*PKP2* mut vs. other desmosomal mut)	p>0.9999
Appendix Figure S2C (*PKP2* mut vs. non desmosomal mut)	p>0.9999
Appendix Figure S2C (*PKP2* mut vs. gene elusive)	p>0.9999
Appendix Figure S3A (Free cholesterol)	p=0.05
Appendix Figure S3A (Triglycerides)	p=0.04
Appendix Figure S3A (Cholesteryl esters)	p=0.143
Appendix Figure S3A (Free fatty acids)	p=0.442
Appendix Figure S3B (ORO/Triglycerides)	p=0.007
Appendix Figure S3B (ORO/Cholesteryl esters)	p=0.1322
Appendix Figure S4A	p=0.005
Appendix Figure S4B (shRNA AM vs. AM+oxLDL)	p=0.05
Appendix Figure S4B (scramble AM vs. AM+oxLDL)	p>0.9999
Appendix Figure S4B (scramble AM vs. shRNA AM)	p=0.001
Appendix Figure S4B (scramble AM+oxLDL vs. shRNA AM+oxLDL)	p=0.010
Appendix Figure S5A (HC AM+rosi vs. ACM AM+rosi)	p=0.01
Appendix Figure S5A (HC GM vs. ACM GM)	p=0.936
Appendix Figure S5A (HC GM vs. HC AM+rosi)	p=0.100
Appendix Figure S5A (ACM GM vs. ACM AM+rosi)	p=0.0002
Appendix Figure S5B (HC GM vs. AM+rosi)	p=0.004
Appendix Figure S5B (ACM GM vs. AM+rosi)	p=0.01
Appendix Figure S5B (HC AM+rosi vs. ACM AM+rosi)	p>0.9999
Appendix Figure S5B (HC GM vs. ACM GM)	p=0.621
Appendix Figure S5C (ACM GM vs. AM+rosi)	p=0.009
Appendix Figure S5C (HC GM vs. AM+rosi)	p=0.927
Appendix Figure S5C (HC AM+rosi vs. ACM AM+rosi)	p=0.212
Appendix Figure S5C (HC GM vs. ACM GM)	p>0.9999
Appendix Figure S6A	p=0.831
Appendix Figure S6B	p=0.0001
Appendix Figure S6C	p=0.701
Appendix Figure S6D	p=0.176
Appendix Figure S6E (RV EF)	p=0.755
Appendix Figure S6E (LV EF)	p=0.550
Appendix Figure S7	p=0.007
Appendix Figure S9B (PPARγ WT CD vs. *Pkp2*+/- CD)	p=0.03
Appendix Figure S9B (PPARγ WT HFD vs. *Pkp2*+/- HFD)	p=0.05
Appendix Figure S9B (PPARγ WT CD vs. HFD)	p=0.051
Appendix Figure S9B (PPARγ *Pkp2*+/- CD vs. HFD)	p=0.080
Appendix Figure S9B (MDA WT HFD vs. *Pkp2*+/- HFD)	p=0.001
Appendix Figure S9B (MDA WT CD vs. *Pkp2*+/- CD)	p=0.018
Appendix Figure S9B (MDA WT CD vs. HFD)	p=0.140
Appendix Figure S9B (MDA *Pkp2*+/- CD vs. HFD)	p=0.007
Appendix Figure S9B (CD36 WT HFD vs. *Pkp2*+/- HFD)	p=0.04
Appendix Figure S9B (CD36 WT CD vs. *Pkp2*+/- CD)	p=0.018
Appendix Figure S9B (CD36 WT CD vs. HFD)	p<0.0001
Appendix Figure S9B (CD36 *Pkp2*+/- CD vs. HFD)	p<0.0001
Appendix Figure S9C (PPARγ)	p=0.0009
Appendix Figure S9C (MDA)	p=0.005
Appendix Figure S9C (CD36)	p<0.0001
Appendix Figure S10B (diastolic area WT CD vs. HFD)	p>0.9999
Appendix Figure S10B (diastolic area *Pkp2*+/- CD vs. HFD)	p=0.116

Appendix Figure S10B (diastolic area *Pkp2*+/- HFD vs. HFD+atorva)	p>0.9999
Appendix Figure S10B (diastolic area *Pkp2*+/- CD vs. HFD+atorva)	p>0.9999
Appendix Figure S10B (diastolic area WT CD vs. *Pkp2*+/- CD)	p>0.9999
Appendix Figure S10B (diastolic area WT HFD vs. *Pkp2*+/- HFD)	p=0.3034
Appendix Figure S10B (wall thickness WT CD vs. HFD)	p>0.9999
Appendix Figure S10B (wall thickness *Pkp2*+/- CD vs. HFD)	p>0.9999
Appendix Figure S10B (wall thickness *Pkp2*+/- HFD vs. HFD+atorva)	p>0.9999
Appendix Figure S10B (wall thickness *Pkp2*+/- CD vs. HFD+atorva)	p>0.9999
Appendix Figure S10B (wall thickness WT CD vs. *Pkp2*+/- CD)	p>0.9999
Appendix Figure S10B (wall thickness WT HFD vs. *Pkp2*+/- HFD)	p>0.9999
Appendix Figure S11A (QRSp *Pkp2*+/- CD vs. HFD)	p=0.0001
Appendix Figure S11A (QRSp WT CD vs. HFD)	p>0.9999
Appendix Figure S11A (QRSp WT CD vs. *Pkp2*+/- CD)	p>0.9999
Appendix Figure S11A (QRSp WT HFD vs. *Pkp2*+/- HFD)	p=0.0001
Appendix Figure S11A (TAD *Pkp2*+/- CD vs. HFD)	p=0.0031
Appendix Figure S11A (TAD WT CD vs. HFD)	p>0.9999
Appendix Figure S11A (TAD WT CD vs. *Pkp2*+/- CD)	p>0.9999
Appendix Figure S11A (TAD WT HFD vs. *Pkp2*+/- HFD)	p=0.0005
Appendix Figure S11A (QRS amplitude *Pkp2*+/- CD vs. HFD)	p=0.0007
Appendix Figure S11A (QRS amplitude WT CD vs. HFD)	p>0.9999
Appendix Figure S11A (QRS amplitude WT CD vs. *Pkp2*+/- CD)	p>0.9999
Appendix Figure S11A (QRS amplitude WT HFD vs. *Pkp2*+/- HFD)	p<0.0001
Appendix Figure S11B (*Pkp2*+/- CD vs. HFD)	p=0.0091
Appendix Figure S11B (WT CD vs. HFD)	p>0.9999
Appendix Figure S11B (WT CD vs. *Pkp2*+/- CD)	p>0.9999
Appendix Figure S11B (WT HFD vs. *Pkp2*+/- HFD)	p=0.095
Appendix Figure S12A (QRSp)	p<0.0001
Appendix Figure S12A (TAD)	p<0.0001
Appendix Figure S12A (QRS amplitude)	p=0.0029
Appendix Figure S12B	p=0.049

Supplemental References:

Cox MG, van der Smagt JJ, Wilde AA, Wiesfeld AC, Atsma DE, Nelen MR, Rodriguez LM, Loh P, Cramer MJ, Doevendans PA, van Tintelen JP, de Bakker JM, Hauer RN (2009) New ECG criteria in arrhythmogenic right ventricular dysplasia/cardiomyopathy. *Circ Arrhythm Electrophysiol* **2:** 524-530

De Lazzari M, Zorzi A, Cipriani A, Susana A, Mastella G, Rizzo A, Rigato I, Bauce B, Giorgi B, Lacognata C, Iliceto S, Corrado D, Perazzolo Marra M (2018) Relationship Between Electrocardiographic Findings and Cardiac Magnetic Resonance Phenotypes in Arrhythmogenic Cardiomyopathy. *Journal of the American Heart Association* **7:** e009855

Fontes MS, van Veen TA, de Bakker JM, van Rijen HV (2012) Functional consequences of abnormal Cx43 expression in the heart. *Biochim Biophys Acta* **1818:** 2020-2029

Grossmann KS, Grund C, Huelsken J, Behrend M, Erdmann B, Franke WW, Birchmeier W (2004) Requirement of plakophilin 2 for heart morphogenesis and cardiac junction formation. *J Cell Biol* **167:** 149-160

Peters S, Trummel M, Koehler B (2008) QRS fragmentation in standard ECG as a diagnostic marker of arrhythmogenic right ventricular dysplasia-cardiomyopathy. *Heart Rhythm* **5:** 1417-1421

Sommariva E, Brambilla S, Carbucicchio C, Gambini E, Meraviglia V, Dello Russo A, Farina FM, Casella M, Catto V, Pontone G, Chiesa M, Stadiotti I, Cogliati E, Paolin A, Ouali Alami N, Preziuso C, d'Amati G, Colombo GI, Rossini A, Capogrossi MC, Tondo C, Pompilio G (2016) Cardiac mesenchymal stromal cells are a source of adipocytes in arrhythmogenic cardiomyopathy. *Eur Heart J* **37:** 1835-1846

Zhang L, Liu L, Kowey PR, Fontaine GH (2014) The electrocardiographic manifestations of arrhythmogenic right ventricular dysplasia. *Curr Cardiol Rev* **10:** 237-245
